# A Bibliometric Analysis of HPV-Positive Oropharyngeal Squamous Cell Carcinoma from 2000 to 2023

**DOI:** 10.3390/pathogens14030289

**Published:** 2025-03-15

**Authors:** Yingying Cui, Wei Li, Binbin Li

**Affiliations:** 1 Department of Oral Pathology, Peking University School and Hospital of Stomatology, National Center for Stomatology, National Clinical Research Center for Oral Diseases, National Engineering Research Center of Oral Biomaterials and Digital Medical Devices, Beijing 100081, China; 2 Research Unit of Precision Pathologic Diagnosis in Tumors of the Oral and Maxillofacial Regions, Chinese Academy of Medical Sciences, Beijing 100081, China

**Keywords:** bibliometric analysis, human papillomavirus, oropharyngeal squamous cell carcinoma

## Abstract

Human-papillomavirus-positive oropharyngeal squamous cell carcinoma (HPV-positive OPSCC) is a distinct disease characterized by unique clinical and molecular features compared to HPV-negative OPSCC. A comprehensive bibliometric analysis of HPV-positive OPSCC research was conducted in this study to identify key trends, research hotspots, and emerging frontiers in the field. Data were retrieved from the Web of Science Core Collection database. The distributions of contributors, including countries, institutions, authors, journals, and cooperative networks related to HPV-positive OPSCC, were analyzed and visualized using VOSviewer 1.6.20, CiteSpace 6.3.R1, and the R package Bibliometrix 4.0.0. In addition, the most influential publications and high-frequency keywords were identified and analyzed to discern key topics in this field. A total of 3895 articles and reviews on HPV-positive OPSCC were identified, involving 106 countries, 620 journals, and 18949 authors. The main contributors include the USA (1908 publications), Johns Hopkins University (310 publications), the journal Head and Neck (320 publications), and Erich M. Sturgis (94 publications). The top three keywords are “survival”, “radiotherapy”, and “p16”. There has been a steadily increasing research interest in HPV-positive OPSCC over the last 23 years. Current studies focus on diagnosis, treatment strategies, prognosis, recurrence, and disease surveillance. This bibliometric analysis highlights key contributors and emerging themes, offering insights for future research directions.

## 1. Introduction

Human papillomavirus (HPV) infection, especially HPV type 16 and 18 (HPV-16/-18), is associated with an increased risk of oropharyngeal squamous cell carcinoma (OPSCC). HPV-positive OPSCC is a distinct entity separate from HPV-negative OPSCC [1], exhibiting distinct clinical and genetic-molecular characteristics. The incidence of HPV-positive OPSCC has increased rapidly over the past two decades, mostly in high-income countries [2,3,4]. The epidemiological statistical reports of HPV-positive OPSCC in Denmark, the United Kingdom, the United States, Thailand, and other countries substantiate this escalating trend [5,6,7]. The significant increase in the incidence rates of HPV-positive OPSCC has posed numerous challenges in its diagnosis, management strategies, and outcome reporting.

Notably, HPV-positive OPSCC tends to have a more favorable prognosis and tends to occur in younger, nonsmoking individuals compared to HPV-negative OPSCC [1,8]. The viral oncoproteins E6 and E7 are the main factors promoting the carcinogenic process of HPV-positive OPSCC, which bind and inactivate the tumor suppressors p53 and the retinoblastoma protein (pRb), respectively [1,9]. Additionally, the functional inactivation of pRb by the viral E7 oncoprotein results in compensatory overexpression of p16 [10], a cyclin-dependent kinase inhibitor that is often used as a surrogate marker for HPV infection [11,12,13]. It is important to distinguish between HPV-positive and HPV-negative OPSCC as this may impact treatment guidelines.

Compared with their HPV-negative counterparts, patients with HPV-positive OPSCC tend to have a better prognosis, as evidenced by the significantly lower rates of both cancer-specific mortality and all-cause mortality, which is partly attributed to a better response to treatment with radiation and chemotherapy [8]. Additionally, HPV-positive patients have been reported to have significantly lower levels of disease recurrence and progression than patients with HPV-negative OPSCC [14]. However, the underlying biological mechanisms responsible for these survival differences are not yet fully understood.

Considering the unique clinical and molecular manifestations as well as favorable therapeutic outcomes of HPV-positive OPSCC, researchers are actively investigating de-escalated treatment strategies for patients with a favorable prognosis, aiming to mitigate treatment-related complications without impairing therapeutic efficacy because although standard treatments are effective in cancer control, they can cause toxicity [15,16]. Sinha, P et al. proposed the avoidance of planned primary bed radiation in T1-T2 HPV-positive OPCC patients with margin-negative resection, as they reported that eliminating planned radiation in these patients did not significantly compromise local control. Instead, it was associated with superior swallowing, as measured by a reduced need for a gastrostomy tube, serving as a surrogate indicator [17]. In another study, Cramer, J. D and his group reported that adjuvant radiation (RT) or chemoradiation (CRT) does not provide a survival benefit for patients with stage I HPV-positive OPSCC with low- or intermediate-risk pathologic features, on the basis of their observational study [18]. There are many other strategies focused on the de-intensified treatment, such as reducing the radiation therapy dose, and the use of less toxic concurrent systemic regimens (such as weekly cisplatin or cetuximab) [19,20,21]. For further investigations, more clinical trials and clinical practices are needed to examine their efficacy in cancer management and complication reduction.

Although HPV status is considered an independent risk factor for the occurrence of HPV-positive OPSCC, the oncogenic pathway that results in malignant transformation and the fundamental mechanism leading to favorable prognosis are not well understood. Further investigation is warranted. Bibliometric analysis is a method that uses mathematical and statistical methods to analyze published studies in specific fields, and it can identify the most influential authors, institutions, countries/regions, as well as the most cited publications and high-frequency keywords [22,23]. This study aims to investigate the research landscape and identify research frontiers in the field of HPV-positive OPSCC through bibliometric analysis, identifying directions for future research regarding the diagnosis, treatment, and prognosis of this specific subset of OPSCC.

## 2. Methods

### 2.1. Data Collection

**Figure 1:**
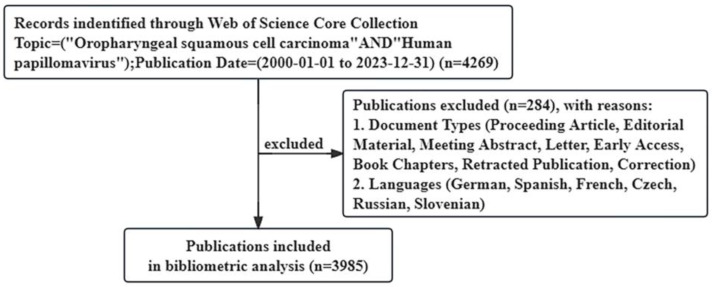
Flow diagram of the publication search process.

The publications for this study were searched and screened on the Web of Science Core Collection (WoSCC), one of the most authoritative databases that contains more than 12,000 top-quality journals and comprehensive data for bibliometric studies [24]. The data needed for this study were retrieved from WoSCC and exported in plain text file format with full records and cited references. The search strategy was “Topic = (Oropharyngeal squamous cell carcinoma AND Human papillomavirus”, and the time span was from 1 January 2000, to 31 December 2023. The document types were limited to articles and review articles, and publications not written in English were excluded. Article includes research papers, brief communications, technical notes, chronologies, full papers, and case reports (presented like full papers) that were published in a journal and/or presented at a symposium or conference. Ultimately, a total of 3985 publications (3347 articles and 638 review articles) were included in the study (Figure 1). The included studies included human studies and preclinical studies.

### 2.2. Data Analysis

Microsoft Office Excel 2021 was used for data organization and statistical table creation in this study. The software VOSviewer 1.6.20, CiteSpace 6.3.R1, and the R package Bibliometrix 4.0.0 are powerful tools for processing and visualizing bibliometric data, each with its own adept analysis scope [25,26,27]. VOSviewer 1.6.20 was employed for keyword co-occurrence plot analysis and co-authorship analysis, which refers to investigating the relatedness of items determined by their number of co-authored documents, involving authors, organizations, and countries. It is also utilized in the citation analysis of publications. CiteSpace 6.3.R1 was used in the dual-map of journal analysis as well as citation bursts analysis of keywords, whereas the R package Bibliometrix 4.0.0 was used to visualize keyword distributions.

## 3. Results

### 3.1. Main Information

**Figure 2:**
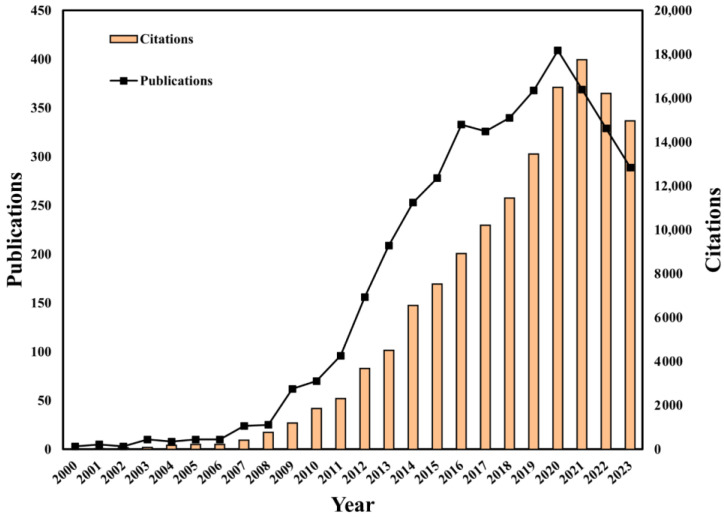
Global publication output and citation trend of HPV-positive OPSCC from 2000 to 2023.

A total of 3895 publications involving HPV-positive OPSCC were included in this study, with a total of 139,311 citations. The number of publications on HPV-positive OPSCC has increased over the past two decades, with a significant growth trend from 2008 to 2020 and peaking in 2020, with 409 articles published. The highest number of citations occurred in 2021, with 17,749 citations accounting for 12.7% of the total citations. The overall information on the articles’ publication trends and citation trends is shown in Figure 2.

### 3.2. Analysis of HPV-Positive OPSCC Research at the National Level

The United States led in the number of HPV-positive OPSCC publications, accounting for 47.9% of the total and 5.9 times more than Germany, which ranked second. The United States was also the most cited country. Notably, nine of the top ten most productive countries were developed countries (Table 1). The 8th, 11th, 12th, 13th, and 14th countries according to the number of citations are Spain, Sweden, Belgium, and Brazil, respectively.

**Figure 3:**
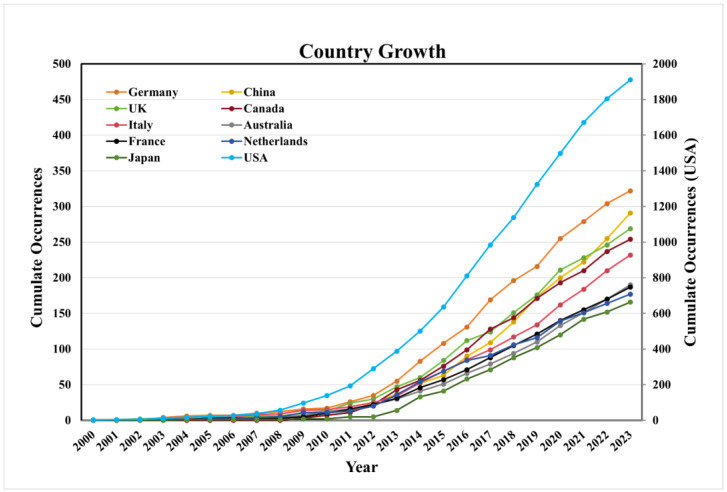
The growth trend of publications on HPV-positive OPSCC in the top 10 productive countries.

As shown in Figure 3, there was a steady growth trend of publications in the top ten productive countries since 2011. The United States has the earliest start in HPV-positive OPSCC research, the fastest growth in the number of papers, and the largest number of published papers, leading global HPV-positive OPSCC research.

**Figure 4:**
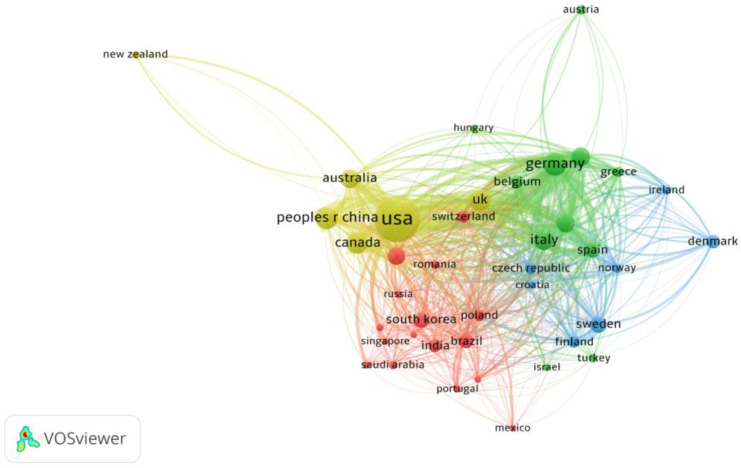
Cooperative relationship between the top 40 most productive countries. (Each node represents an individual country, and the node size represents the number of publications. The width of the lines indicates the link strength.)

Collaboration with HPV-positive OPSCC at the country level was analyzed and visualized (Figure 4). The collaboration network analysis of the top 40 countries in terms of the number of publications on HPV-positive OPSCC revealed that these countries were divided into four clusters on the basis of the closeness of collaboration. The United States leads international collaboration in HPV-positive OPSCC research, followed by Germany, the United Kingdom, Italy, and Spain, with the closest cooperative relationship with China (link strength of 99) (Figure 4). Furthermore, robust collaborative ties are evident among the United States, China, Canada, and Germany, indirectly reflecting the increasing prevalence of HPV-positive OPSCC in economically developed regions.

### 3.3. Institution Analysis

Among the approximately 4000 institutions participating in the research of HPV-positive OPSCC, Johns Hopkins University emerged as both the most productive and the most frequently cited organization in HPV-positive OPSCC research, followed closely by the University of Texas MD Anderson Cancer Center. The latter is noTa for hosting the most-cited author and publishing the most highly cited research article on HPV-positive OPSCC. Among the top ten institutions by publication count in this field, the majority are based in the United States (Table 2).

### 3.4. Author Analysis

According to the exported publication records, the number of researchers devoted to the field of HPV-positive OPSCC reached a remarkable 18949, and these researchers published 34674 articles in this field in total, with 1370618 citations. Erich M. Sturgis, a scholar from Baylor College of Medicine, was the most productive author, with 94 articles on HPV-positive OPSCC published, and his work was cited 5317 times as of 2023. The most-cited author is Maura L. Gillison, a scholar from the University of Texas MD Anderson Cancer Center whose H-index is 87. She has 42 publications in the field of HPV-positive OPSCC, with 16545 citations in total (Table 3).

### 3.5. Journal Analysis

A total of 621 journals were included in the bibliometric study. The top ten journals ranked by the number of publications on HPV-positive OPSCC were screened out and listed in Table 4. *Head and Neck*, published by Wiley, was the most productive publication source in this field, whose IF and CiteScore are 2.9 and 6.9, respectively. Meanwhile, the journal with the most citations was the *Journal of Clinical Oncology*, with 12705 citations across 47 articles. It was ranked 15th in terms of the number of documents published. Published by ASCO, the journal has an IF and CiteScore of 45.3 and 39.6, respectively.

**Figure 5:**
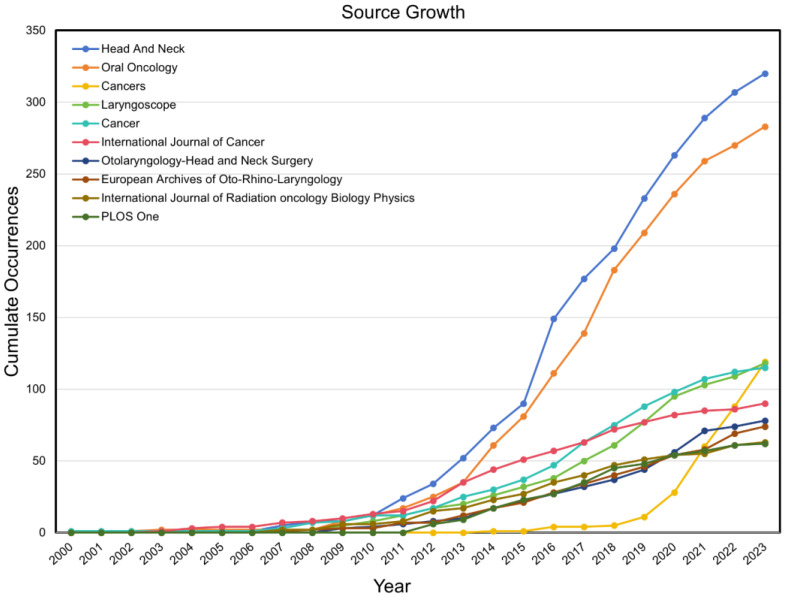
The growth trend of publications in the top 10 productive journals on HPV-positive OPSCC.

*Head and Neck* and *Oral Oncology* demonstrated significantly higher publication volumes of articles compared to the others, collectively accounting for 15.1% of the total publications included in the study, with 8.0% and 7.1%, respectively, for each journal. In addition, the two journals above had the most significant rate of growth in the number of articles published on HPV-positive OPSCC research, with the cumulative number of publications in 2023 being approximately six times greater than that a decade ago (Figure 5). *Cancers* has experienced a significate increase in the number of publications since 2019, and the cumulative number of publications in 2023 was approximately ten times greater than that in 2019. It ranked third in terms of the number of publications in the field of HPV-positive OPSCC (Table 4).

**Figure 6:**
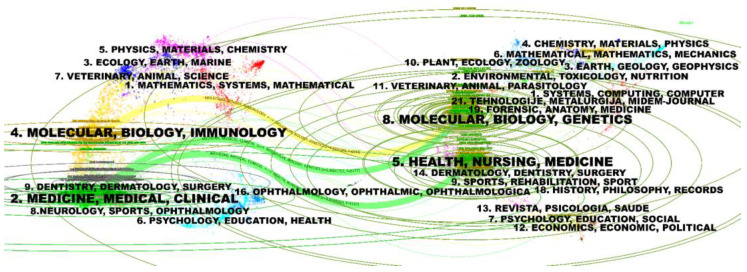
Dual-map overlay and corresponding disciplines. (The citing journals are on the left, the cited journals are on the right, and the colored path represents the citation relationship).

The dual-map of journals, generated by CiteSpace 6.3.R1, represents the main citation paths from the cited publications and the citing publications on HPV-positive OPSCC. It turned out that there were three main citation paths, colored in yellow and green. The studies published in molecular/biology/immunology and medicine/medical/clinical journals mainly cited publications in molecular/biology/genetics journals. In addition, the studies published in medicine/medical/clinical journals also had a strong connection with publications in health/nursing/medicine journals (Figure 6). The citing studies on the left to some degree demonstrated the frontiers, research trend, as well as hotpots in the field of HPV-positive OPSCC. Studies at the forefront were related to immune and clinical research on OPSCC.

### 3.6. Top 10 Publications

The top ten papers with the highest number of citations were recognized and extracted from the 3895 searched publications (Table 5). The most influential article, titled “Human Papillomavirus and Survival of Patients with Oropharyngeal Cancer”, was written by K. Kian Ang and published in *The New England Journal of Medicine* in 2010.

### 3.7. High-Frequency Keyword Analysis

**Figure 7:**
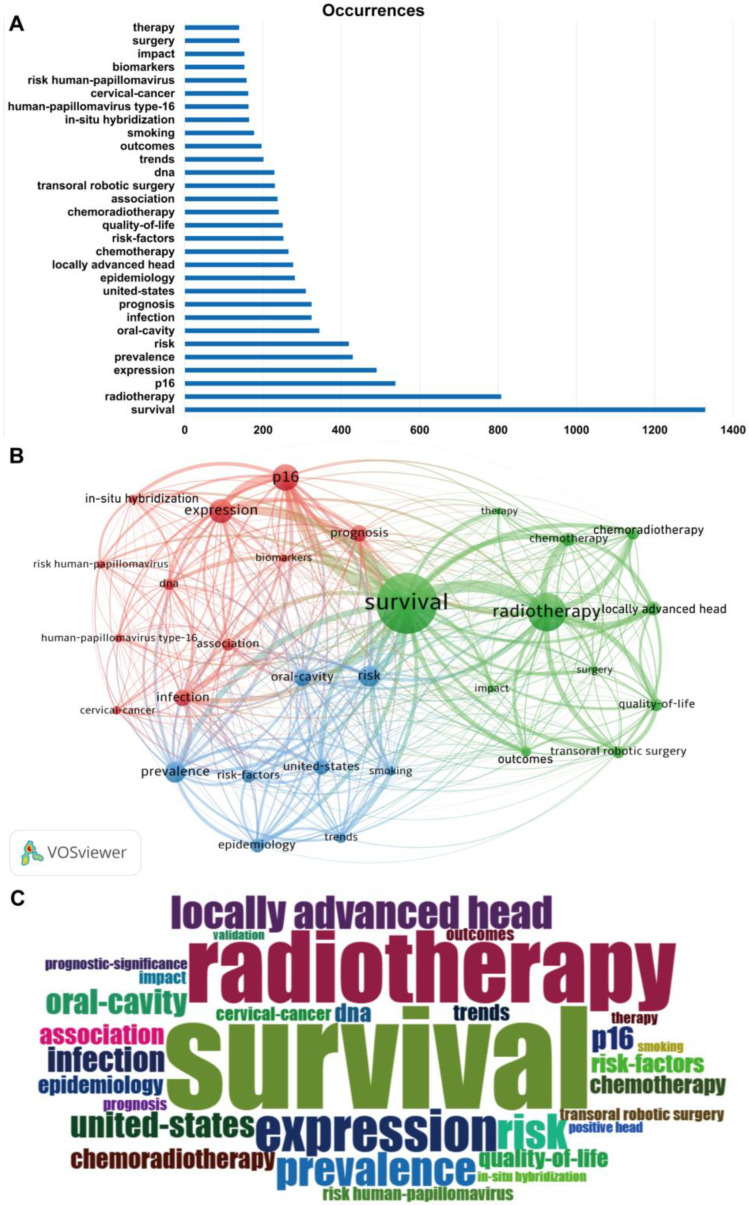
The top 30 high-frequency keywords associated with HPV-positive OPSCC. (**A**) The bar graph illustrates the frequency of occurrence. (**B**) Word co-occurrence plot. Each node in the figure represents a keyword, and the color of the word indicates its cluster group. (**C**) Word cloud plot.

Keywords are the condensation and refinement of the core content of publications, and the high frequency of keywords can reflect the research hotspots in HPV-positive OPSCC research. After excluding terms synonymous with HPV and OPSCC, the top 30 keywords based on frequency included survival (frequency of 1329), radiotherapy (808), locally advanced head (277), chemotherapy (265), quality-of life (250), chemoradiotherapy (240), transoral robotic surgery (230), outcomes (196), impact (152), surgery (140), therapy (139), which were related to the treatment and prognosis of HPV-positive OPSCC (green cluster). Another keyword cluster (red cluster) included p16 (frequency of 538), expression (490), infection (324), prognosis (324), association (237), dna (229), in-situ hybridization (164), human-papillomavirus type-16 (163), cervical cancer (162), risk human-papillomavirus (158), biomarkers (152), relevant to basic research and biological characteristics of HPV-positive OPSCC. Prevalence (429), risk (419), oral-cavity (344), united-states (309), epidemiology (281), risk-factors (252), trends (201), and smoking (177) were combined in a cluster (blue cluster) involving the epidemiology and risk factors (Figure 7A,B). A word cloud plot of the top 30 high-frequency keywords on HPV-positive OPSCC was generated and visualized by the R package Bibliometrix 4.3.3 (Figure 7 C). 

**Figure 8:**
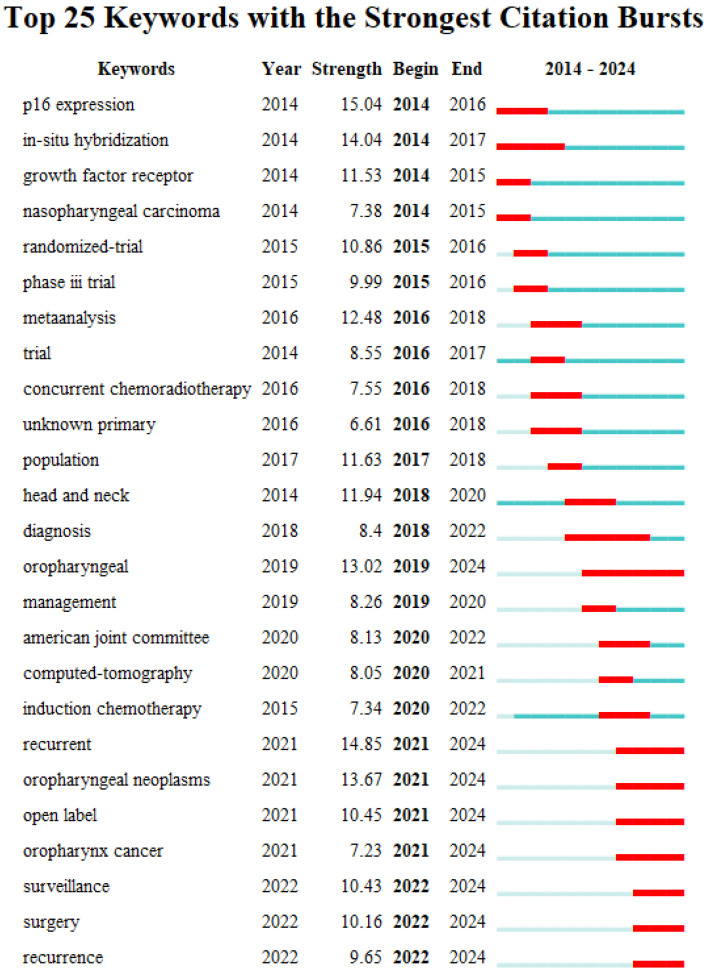
Burst graph of keywords. Red lines indicate years of frequent appearances, and green lines indicate years of fewer appearances.

Burst words are terms that appear with a large change in frequency in a relatively short period of time. The analysis of burst words (citation burst analysis) can also provide an aspect of research hotspots in a specific time period and reveal the research frontiers and development trends of a certain research field. Figure 8 shows the mapping of the top 25 emerging keywords in HPV-positive OPSCC research, including the burst words, citation strength, and the time of citation burst. Red lines indicate years of frequent appearances, and green lines indicate years of fewer appearances.

In terms of citation strength, the term "p16" has the highest intensity, reaching up to 15.04. This keyword represents an important aspect of early HPV-positive OPSCC research and has become a significant research frontier hotspot. Studies have found that p16 is an independent prognostic factor for OPSCC and serves as a good surrogate for HPV status in OPSCC. Additionally, the terms "recurrent" and "in-situ hybridization" also have high burst strengths, at 14.85 and 14.04, respectively, indicating that they are important frontier areas in HPV-positive OPSCC research. Overall, the molecular characteristics and disease prognosis of HPV-positive OPSCC, especially precise diagnostic methods for HPV status in OPSCC and tumor recurrence, have become core frontier hotspots in HPV-positive OPSCC research, attracting widespread attention from researchers.

Regarding the time of citation burst, research before 2016 mainly focused on the molecular characteristics of OPSCC, discovering a strong agreement between expression of p16 and tumor HPV status in OPSCC and better prognosis for p16-positive patients. From 2017 to 2020, research concentrated on the clinical management and treatment of HPV-positive OPSCC, with clinical trials exploring treatment de-escalation strategies. After 2021, continuous monitoring post-treatment and postoperative recurrence in HPV-positive OPSCC have become research hotspots.

## 4. Discussion

### 4.1. General Information

Significant efforts have been made in the research on HPV-positive OPSCC over the past two decades. In this study, we included 3895 published articles and reviews, engaging with 106 countries, 620 journals, and 18949 authors. Johns Hopkins University was the most productive as well as most cited organization, followed by the University of Texas MD Anderson Cancer Center, which is affiliated with the most-cited author and published the most-cited research article with respect to HPV-positive OPSCC. Among the top ten institutions ranked by the number of publications on HPV-positive OPSCC, the majority were located in the United States, indicating it was the leading country in conducting relevant research. Strong cooperation connections were observed between the United States, China, Canada, and Germany. In terms of authors, Erich M. Sturgis, a professor from Baylor College of Medicine, has made great contributions to the field of HPV-positive OPSCC, publishing the largest number of research articles in this field. His first research article on HPV-positive OPSCC was published in *Clinical Cancer Research* in 2003, whose title was “Human papillomavirus type 16 infection and squamous cell carcinoma of the head and neck in never-smokers: A matched pair analysis”, concluding that HPV-16 infection is associated with a significantly increased risk for OPSCC [28]. His most highly cited paper titled “Radiotherapy plus cetuximab or cisplatin in human-papillomavirus-positive oropharyngeal cancer (NRG Oncology RTOG 1016): a randomised, multicentre, non-inferiority trial” was published in Lancet in 2019 and focused on the treatment strategy of HPV-positive OPSCC. His team conducted a clinical trial in which patients with HPV-positive OPSCC were randomly assigned into two groups: one receiving radiotherapy plus cetuximab and the other receiving radiotherapy plus cisplatin (the standard of care for those eligible with HPV-positive OPSCC). By comparing the overall survival and progression-free survival rates of the two treatment strategies, they concluded that the radiotherapy plus cetuximab strategy was more effective for the treatment of HPV-positive OPSCC [21]. Maura L. Gillison, from the University of Texas MD Anderson Cancer Center, was the most-cited author, and her most-cited publication was the one for which she was the corresponding author called “Human Papillomavirus and Survival of Patients with Oropharyngeal Cancer”. This article was also the most cited article in the field of HPV-positive OPSCC research [13]. It is very well-founded to say that Maura L. Gillison and her research team have made significant contributions to the field of HPV-positive OPSCC research, greatly advancing its in-depth research. Her research group performed a retrospective analysis of the association between HPV status and survival among HPV-positive OPSCC patients and concluded that tumor HPV status is a strong and independent prognostic factor for survival among patients with OPSCC. Their research data indicated that overall survival was primarily determined by the HPV status of the tumor, followed by tobacco smoking pack-years, the nodal stage for HPV-positive tumors, and the tumor stage for HPV-negative tumors. In addition, they reported that there was no significant difference in the 30-day or 3-year rates of overall survival between the two HPV-positive OPSCC patient groups who had received the accelerated-fractionation radiotherapy or the standard-fractionation radiotherapy, and the groups also did not differ significantly with respect to progression-free survival or the pattern of relapse. In addition to these findings, they also assessed the sensitivity of methods for detecting the HPV status, including the HPV-16 in situ hybridization assay and p16 expression assay, and proposed that the p16 expression status was a very good surrogate for the tumor HPV status. The findings and conclusions therein have had a considerable impact on subsequent research. The 8th edition of the American Joint Commission on Cancer (AJCC8) has divided OPSCC into HPV-mediated (p16+) and non-HPV-mediated (p16−) OPSCC on the basis of the status of p16 overexpression and has proposed a separate clinical and pathological staging system for HPV-positive OPSCC compared with a conventional primary oral squamous cell carcinoma or neck metastasis of similar size [29].

### 4.2. Hotspots and Frontiers

#### 4.2.1. Epidemiology

Globally, the incidence of HPV-positive OPSCC has increased rapidly over the past two decades, especially in younger men [2,30]. Developed countries with high-income populations, such as the North American, Brazil, Australian, and European countries, tend to report a greater proportion of HPV infection in OPSCC [31,32,33,34,35,36,37,38]. In the United States, the prevalence of HPV-positive OPSCC increased rapidly from 1995 to 2019 among all sex and race groups. Moreover, the incidence of HPV-positive OPSCC in men has surpassed that of cervical cancer in women, which is closely associated with HPV. In addition, risky sexual behaviors such as having two or more lifetime sex partners and oral sex were consistent with HPV exposure, and were established as risk factors for HPV-positive OPSCC, suggesting directions for future disease prevention [39,40,41,42]. However, a study conducted in Germany reported different results, which by showing a low prevalence of risky sexual behaviors in OPSCC patients, confirmed findings from other European studies that differed substantially from North American case–control studies [43].

There were fewer statistical reports about developing countries than high-income countries. In Malaysia, the prevalence of HPV-positive OPSCC had an upward trend but was not as high as those of some developed countries. The same tendency was observed in southern Thailand [44,45]. The increase in HPV-positive OPSCC highlights the urgent need for public health interventions. More attention should be given to disease surveillance, treatment strategy studies, and prevention measures.

#### 4.2.2. HPV Status Detection Methods

The cyclin-dependent kinase inhibitor p16, a known biomarker of HPV oncoprotein function, is induced as a consequence of pRb inactivation by the HPV E7 oncoprotein. The expression of p16 in tumors is strongly consistent with the tumor’s HPV status, as examined by in situ hybridization, and is now routinely used as a satisfactory surrogate for tumor HPV status [13,42]. The AJCC 8th edition uses the surrogate marker p16 to stratify for HPV-positive OPSCC. Compared with other HPV detection methods, p16 IHC yields the best stratification of outcomes [46]. However, whether p16 alone can be used to determine the HPV status is questionable because a mutation in pRb can also lead to the overexpress of p16. All p16 immunohistology (IHC)-positive OPSCCs should be considered for retesting using HPV mRNA in situ hybridization (ISH) to verify transcriptionally active HPV [47]. Furthermore, the criteria for defining p16-positive vary among studies [11,48,49]. Defining p16 overexpression as showing a strong and diffuse nuclear and cytoplasmic staining in at least 70% or more of the tumor cells may achieve the highest correlation between p16-IHC and HPV results [11]. However, further studies on the efficiency of p16 overexpression in recognizing HPV-positive tumors are needed.

#### 4.2.3. Treatment Strategy

Patients with HPV-positive OPSCC tend to have a more favorable prognosis and are more sensitive to standard surgery, radiotherapy, and chemotherapy than those with HPV-negative OPSCC. Despite the satisfactory cancer control provided by standard treatments, they can result in both acute and late toxicities, which has prompted the exploration of de-escalated treatment strategies [15,42].

Due to the deep anatomical location of the oropharynx, the lip-splitting mandibulotomy has been the traditional approach for resecting large oropharyngeal tumors in order to obtain adequate margin visualization and free flap reconstruction of surgical defects. However, it is also associated with significant facial scarring and delayed recovery of oral function [50]. Transoral robotic surgery (TORS), developed by Weinstein et al. in 2007 [51], is a minimally invasive surgical technique, which improves visualization in the area of the oropharynx, and is beneficial for improving operative precision and minimizing tissue disruption, along with eliminating surgical incision recovery time [52]. There are several studies that demonstrate the feasibility of performing transoral resection of small HPV-positive OPSCC tumors using a robot [52,53,54], and it was revealed that TORS provides favorable survival outcomes and typically results in superior swallowing function posttreatment compared with other therapeutic modalities in HPV-positive OPSCC [55]. Marius Meldgaard Justesen and his group conducted a consecutive single-institution study from 2013 to 2020 and offered TORS to patients staged with cT1-2, N0-1, and M0 OPSCC. They ultimately included 153 patients who were treated with curative intent by TORS from 2013 to 2020 and assessed their overall survival (OS), recurrence-free survival (RFS), recurrence patterns, and ultimate failure rate (UFR). They reported that excellent survival and disease control were obtained with TORS + neck dissection in this cohort, despite fewer applications of adjuvant therapy than other TORS centers, implying that TORS without adjuvant therapy can be successfully applied in the treatment of early-stage OPSCC. However, the safety distance of the cutoff margin needs to be investigated and examined [54,56].

Radiation (RT) deintensification is a promising approach with ample literature [16,20,57,58]. Bhishamjit S. Chera and his colleagues conducted a clinical study in which patients with HPV-positive OPSCC were treated with intensity-modulated radiotherapy (IMRT) at a dose of 60 grays, combined with weekly intravenous cisplatin at a lower dose of 30 mg/m^2^. The results suggested that, compared with standard therapies, this reduced-intensity therapy not only preserved patients’ quality of life more effectively, but also resulted in excellent 3-year tumor control and survival rates [59,60].

Cisplatin, a DNA intercalating agent that targets rapidly replicating cells, is the most widely used chemotherapeutic agent with the best prognostic outcome [61], but its clinical use is limited by severe, dose-dependent toxic side effects, such as oropharyngeal mucosal inflammation, post-treatment renal toxicity, and neurotoxicity. When combined with radiotherapy, it also elevates the risk of severe dysphagia and progression-free mortality. Consequently, scholars are exploring the possibility of reducing cisplatin dose or seeking alternative drugs [62]. Cetuximab is a monoclonal antibody designed to target the growth factor receptor (EGFR) extracellular ligand binding domain and is used in anti-epidermal EGFR therapies [63]. Several studies have compared cetuximab combined with standard dose radiation therapy with cisplatin-based chemoradiation in HPV-positive OPSCC patients. One such study, RTOG 1016, was a randomized, multicenter, non-inferiority trial conducted across 182 health-care centers in the USA and Canada. Patients were randomly assigned into two groups: one received radiotherapy plus cetuximab, while the other received radiotherapy plus cisplatin. The results indicated that, compared to radiotherapy plus cisplatin, radiotherapy plus cetuximab was associated with inferior overall survival and progression-free survival in patients with HPV-positive OPSCC [21]. However, the anther clinical trial conducted by Hisham Mehanna provided different insights. These findings suggest that, compared with the standard cisplatin regimen, cetuximab has no benefit in terms of reduced toxicity and instead is associated with a significant decrease in tumor control. This trial reinforced the notion that cisplatin and radiotherapy should be used as the standard of care for HPV-positive low-risk patients who are able to tolerate cisplatin [64]. The contrasting results from these trials indicate that, as of now, de-escalated treatment strategies are not yet suitable for routine application outside of clinical trials for patients with HPV-positive OPSCC.

Multiple studies indicate that immunotherapy can improve the overall survival rate and response rate in patients with HPV-positive head and neck cancers [65]. Currently, immunotherapy drugs that have been approved for the clinical treatment of HPV-positive malignant tumors include PD-1 inhibitors such as nivolumab and pembrolizumab, as well as PD-L1 inhibitors such as avelumab and durvalumab. These medications enhance the immune system’s ability to attack tumors by blocking the PD-1/PD-L1 signaling pathway, thereby improving the overall survival rate and response rate of patients [66]. KEYNOTE-040 was a randomized phase III clinical trial that included HPV-positive OPSCC patients with disease recurrence or progression within 3 to 6 months of platinum chemotherapy, in which patients were randomly allocated into two groups, accepting pembrolizumab or standard care (methotrexate, docetaxel, or cetuximab). The group treated with pembrolizumab experienced a lower incidence of adverse events of Grade 3 or higher and had a lower mortality rate [67]. As an emerging therapeutic approach, the benefits of immunotherapy relative to other treatment modalities require further investigation through clinical trials.

#### 4.2.4. Prevention

High-risk HPV subtypes have been shown to be the main risk factors for cervical cancer, HPV-positive OPSCC, and other HPV-positive cancers. Since the introduction of the HPV vaccine in 2015, it has been widely promoted and is now available in many countries to help prevent cervical cancer in women [68]. Reports suggest that the vaccine may also have a potential role in preventing HPV-positive OPSCC. Furthermore, the HPV vaccine is being considered for pangender availability to prevent this type of cancer [69,70,71]. There are some ongoing clinical trials assessing the efficacy of the vaccine in preventing HPV-positive OPSCC [72]. Unfortunately, sufficient data are lacking due to the relatively recent implementation of the HPV vaccine and the late development of HPV-positive OPSCC, which typically affects individuals with a mean age of 40-60 [68,70]. The preventative effect of the vaccine against HPV-positive OPSCC will become clearer as more results are obtained and reported to the public in the future. In addition, NCT02426892 was a phase 2 clinical trial investigating the role of ISA 101, a therapeutic HPV vaccine targeting E6 and E7, in promoting the efficacy of nivolumab, an anti-PD-1 immune checkpoint antibody in the treatment of patients with incurable HPV-positive OPSCC. The overall response rate (33%) and median overall survival (17.5 months) improved in patients who received both nivolumab and ISA 101 compared with PD-1 inhibition alone in similar patients, which confirmed that the contribution of HPV-16 vaccination to the tumoricidal effects of PD-1 inhibition warrants further study [73].

### 4.3. Limitations

There are several limitations inherent to this study. Although the publications for this study were searched and screened in the WoSCC, a database renowned for its authority, there remains a possibility that some relevant HPV-positive OPSCC publications which were not recorded in the WoSCC were not included in our analysis. Furthermore, by focusing solely on English-language publications for this bibliometric study, some important research findings from non-English sources may have been overlooked.

## 5. Conclusions

This study provides a thorough overview of the research landscape for HPV-positive OPSCC from 2000 to 2023 using information visualization analysis, including publication outputs, authoritative countries, institutions, authors, and journals. The United States was the leading contributor to HPV-positive OPSCC research, followed by Germany, China, and the UK. Johns Hopkins University emerged as both the most productive institution and the most frequently cited institution. Erich M. Sturgis, a scholar from Baylor College of Medicine, was the most productive author and the most-cited author was *Maura* L. Gillison, a scholar from the University of Texas MD Anderson Cancer Center. *Head and Neck*, published by Wiley, was the most productive publication source. Moreover, the journal with the most citations was Journal of Clinical Oncology, published by ASCO. The most influential article, titled “Human Papillomavirus and Survival of Patients with Oropharyngeal Cancer”, was written by K. Kian Ang and published in The New England Journal of Medicine in 2010. Current studies on HPV-positive OPSCC have focused on its diagnosis, treatment strategies, prognosis, recurrence, and disease surveillance. The analysis highlights the need for improved diagnostic methods and identifies a critical need for further clinical trials to strengthen the evidence for treatment strategies, especially de-escalation approaches. These discoveries are instrumental in charting future research directions and therapeutic advancements in HPV-positive OPSCC management.

## Figures and Tables

**Table 1:** The top 10 productive countries according to the number of publications related to HPV-positive OPSCC.

Rank	Country	Documents	Citations	SCPs ^1^	MCPs ^2^	MCP Ratio ^3^	Rank based on Citations
1	USA	1908	91,042	1427	481	0.252	1
2	Germany	322	12,710	146	176	0.547	3
3	China	291	5260	149	142	0.488	10
4	UK	269	11,130	111	158	0.587	4
5	Canada	254	18,372	120	134	0.528	2
6	Italy	231	6791	124	107	0.463	7
7	Australia	190	5532	103	87	0.458	9
8	France	187	9836	73	114	0.61	5
9	Netherlands	177	9632	78	99	0.559	6
10	Japan	166	2631	121	45	0.271	15

^1^ SCPs: single-country publications; ^2^ MCPs: multiple-country publications; ^3^ MCP Ratio = MCPs/documents.

**Table 2:** The 10 most productive institutions in the study of HPV-positive OPSCC.

Rank	Institution	Documents	Total Citations	Country	Average Citations per Document
1	Johns Hopkins University	310	36,106	USA	116.47
2	The University of Texas MD Anderson Cancer Center	207	16,003	USA	77.31
3	Washington University	129	7226	USA	56.02
4	Harvard University	126	4615	USA	36.63
5	University Of Michigan	107	5271	USA	49.26
6	University of Pittsburgh	101	5103	USA	50.52
7	Mayo Clinic	99	2867	USA	28.96
8	Karolinska Institutet	98	3786	Sweden	38.63
9	German Cancer Consortium (DKTK)	96	5758	Germany	59.98
10	University of Toronto	87	6716	Canada	77.20

**Table 3:** The 10 most productive authors in the field of HPV-positive OPSCC.

Rank	Author	Documents	Citations	H-Index	Affiliation
1	Erich M. Sturgis	94	5317	74	Baylor College of Medicine
2	Carole Fakhry	87	7139	48	Johns Hopkins University School of Medicine
3	Tina Dalianis	59	2550	49	Karolinska Institutet
4	Guojun Li	59	1428	39	University of Texas School of Public Health
5	James S. Lewis Jr	57	3382	54	Vanderbilt University Medical Center
6	Robert L. Ferris	51	2366	94	University of Pittsburgh Medical Center
7	William H. Westra	51	12,779	114	Icahn School of Medicine at Mount Sinai
8	Brian O’Sullivan	48	4967	99	University of Toronto Faculty of Medicine
9	Qingyi Wei	48	1278	83	Duke University School of Medicine
10	Gypsyamber D’Souza	46	5808	56	Johns Hopkins Bloomberg School of Public Health

**Table 4:** The ten most productive journals that published manuscripts about HPV-positive OPSCC.

Rank	Journal	Documents	Citations	IF (2022)	CiteScore	Publisher
1	*Head and Neck*	320	7585	2.9	6.9	Wiley
2	*Oral Oncology*	283	9317	4.8	8.6	Elsevier
3	*Cancers*	119	1172	5.2	7.4	MDPI
4	*Laryngoscope*	118	2692	2.6	5.7	Wiley
5	*Cancer*	115	6419	6.2	12.2	Wiley
6	*International Journal of Cancer*	90	6838	6.4	15.2	Wiley
7	*Otolaryngology-Head and Neck Surgery*	78	1293	3.4	7.2	Sage
8	*European Archives of Oto-Rhino-Laryngology*	74	985	2.6	5.0	Springer
9	*International Journal of Radiation oncology Biology Physics*	63	2605	7.0	11.0	Elsevier
10	*PLOS ONE*	62	1329	3.7	6.0	PLOS
15	*Journal of Clinical Oncology*	47	12,705	45.3	39.6	ASCO

**Table 5:** The top 10 papers with the highest number of citations on HPV-positive OPSCC.

Rank	Title	Journal	First Author	Total Citations	Year
1	Human Papillomavirus and Survival of Patients with Oropharyngeal Cancer	*The New England Journal of Medicine*	K. Kian Ang	4714	2010
2	Evidence for a Causal Association Between Human Papillomavirus and a Subset of Head and Neck Cancers	*Journal of the National Cancer Institute*	Maura L. Gillison	2262	2000
3	Case–Control Study of Human Papillomavirus and Oropharyngeal Cancer	*The New England Journal of Medicine*	Gypsyamber D’Souza	1913	2007
4	Human Papillomavirus Types in Head and Neck Squamous Cell Carcinomas Worldwide: A Systematic Review	*Cancer Epidemiology, Biomarkers & Prevention*	Aimee R. Kreimer	1520	2005
5	HPV-associated head and neck cancer: a virus-related cancer epidemic	*The Lancet Oncology*	Shanthi Marur	1314	2010
6	Incidence Trends for Human Papillomavirus–Related and –Unrelated Oral Squamous Cell Carcinomas in the United States	*Journal of Clinical Oncology*	Anil K. Chaturvedi	1203	2008
7	Head and Neck Cancers—Major Changes in the American Joint Committee on Cancer Eighth Edition Cancer Staging Manual	*CA-A Cancer Journal for Clinicians*	William M. Lydiatt	976	2017
8	Worldwide Trends in Incidence Rates for Oral Cavity and Oropharyngeal Cancers	*Journal of Clinical Oncology*	Anil K. Chaturvedi	916	2013
9	The molecular landscape of head and neck cancer	*Nature Reviews Cancer*	C. René Leemans	784	2018
10	Epidemiology of Human Papillomavirus–Positive Head and Neck Squamous Cell Carcinoma	*Journal of Clinical Oncology*	Maura L. Gillison	769	2015

## Data Availability

The datasets generated and/or analyzed during the current study are available from the corresponding author on reasonable request.

## Abbreviations

HPV	human papillomavirus
OPSCC HPV-positive OPSCC	oropharyngeal squamous cell carcinoma human-papillomavirus-positive oropharyngeal squamous cell carcinoma
pRb	retinoblastoma protein
RT CRT WoSCC AJCC8 IHC ISH TORS	radiation chemoradiation Web of Science Core Collection the 8th edition of the American Joint Commission on Cancer immunohistology in situ hybridization transoral robotic surgery
